# A Robust Intelligent CNN Model Enhanced with Gabor-Based Feature Extraction, SMOTE Balancing, and Adam Optimization for Multi-Grade Diabetic Retinopathy Classification

**DOI:** 10.3390/jimaging12050188

**Published:** 2026-04-27

**Authors:** Asri Mulyani, Muljono, Purwanto, Moch Arief Soeleman

**Affiliations:** 1 Faculty of Computer Science, Universitas Dian Nuswantoro, Semarang 50131, Indonesia; p41202300049@mhs.dinus.ac.id (A.M.); purwanto@dsn.dinus.ac.id (P.); arief22208@gmail.com (M.A.S.); 2 Department of Computer Science, Institut Teknologi Garut, Garut 44151, Indonesia

**Keywords:** deep learning, diabetic retinopathy, feature extraction, Gabor filter, medical image, classification, intelligent decision support

## Abstract

Diabetic retinopathy (DR) is a leading cause of vision impairment and permanent blindness worldwide, requiring accurate and automated systems for multi-grade severity classification. However, standard Convolutional Neural Networks (CNNs) often struggle to capture fine, high-frequency microvascular patterns critical for diagnosis. This study proposes a Robust Intelligent CNN Model (RICNN) that integrates Gabor-based feature extraction with deep learning to improve DR classification. Specifically, Gabor filters are applied during preprocessing to extract orientation- and frequency-sensitive texture features, which are transformed into feature maps and concatenated with CNN feature representations at the fully connected layer (feature-level fusion). The model also incorporates the Synthetic Minority Oversampling Technique (SMOTE) for data balancing and the Adam optimizer for efficient convergence. This integration enhances sensitivity to microvascular structures such as microaneurysms and hemorrhages. The proposed RICNN was evaluated on the Messidor dataset (1200 images) across four severity levels: Mild, Moderate, Severe, and Proliferative DR. The model achieved an accuracy of 89%, a precision of 88.75%, a recall of 89%, and an F1-score of 89%, with AUCs of 97% for Severe DR and 99% for Proliferative DR. Comparative analysis confirms that the proposed texture-aware Gabor enhancement significantly outperforms LBP and Color Histogram approaches, indicating its potential for reliable clinical decision support.

## 1. Introduction

According to data from the World Health Organization (WHO), diabetic retinopathy (DR) is a leading cause of vision impairment and blindness worldwide [1]. It is estimated that approximately 10% of the global population has diabetes, and approximately one in five individuals has diabetic retinopathy [2]. It is a chronic, progressive retinal disorder that evolves through four clinical stages: Mild, Moderate, Severe Nonproliferative Retinopathy, and Proliferative Diabetic Retinopathy [3,4,5]. Diabetic retinopathy can be diagnosed through fundus imaging, which is an image obtained with a fundus camera, a confocal scanning laser ophthalmoscope, or a near-infrared scanning laser ophthalmoscope [6]. The increasing prevalence of diabetes, coupled with the limited number of retinal specialists, has created a bottleneck in diagnosis. Accurate multi-grade classification is clinically vital because the treatment window narrows significantly in advanced stages, yet manual screening remains labor-intensive and prone to inter-observer variability.

One of the critical stages in developing an image classification system is feature extraction, which converts raw images into compact numerical representations. Commonly used feature extraction methods include the Color Histogram, Edge Detection, Histogram of Oriented Gradients (HOG), Gabor filter, Gray Level Co-occurrence Matrix (GLCM), and Local Binary Pattern (LBP) [7,8,9,10]. Several studies have applied these techniques to fundus images; for instance, Gabor filters combined with adaptive histogram equalization achieved 98.1% accuracy [11], while a single Gabor filter approach yielded 78% accuracy [12]. Similarly, comparisons of LBP and GLCM using support vector machines have shown accuracies between 73% and 74% [13].

In addition to manual methods, deep learning—specifically Convolutional Neural Networks (CNNs)—has become a dominant approach in medical image classification due to its ability to automatically extract features and utilize transfer learning to overcome data limitations [14,15,16]. In the context of diabetic retinopathy (DR), Convolutional Neural Network (CNN)-based models have been widely adopted for automated screening and diagnosis, demonstrating strong performance in analyzing retinal fundus images. Several studies have shown that integrating CNN with image enhancement or optimization techniques can further improve detection accuracy [17,18] and lesion classification [19,20]. Furthermore, hybrid approaches, such as combining CNN with SAJOA for blood vessel segmentation, have also been proposed [21].

To enhance model robustness, combining manual feature extraction with CNNs has been explored in various domains. For example, CNNs integrated with Gabor filters have been used for steganography detection [22], hand gesture recognition [23], general image classification [24], and urban map damage detection [25]. Similarly, the hybridization of CNNs with LBP has been widely studied for melanoma classification [26], oral cancer detection [27], and bearing failure detection [28]. Meanwhile, the integration of Color Histogram analysis with CNNs has been applied to skin lesion classification [29].

However, a critical research gap remains. Although various studies have utilized feature extraction and CNN-based methods, most DR research still relies on standard convolutional architectures without in-depth exploration of domain-specific feature integration techniques. Existing hybrid approaches typically combine CNNs with generic handcrafted features, such as Local Binary Pattern (LBP) or color-based descriptors, but they are not specifically designed to effectively capture high-frequency microvascular patterns, which are important indicators in DR diagnosis.

Furthermore, previous studies often rely on a single feature type or limited feature combinations without comprehensively evaluating the impact of feature integration on multi-level classification performance. The issue of class imbalance is also frequently overlooked, leading to low sensitivity in detecting more advanced, clinically critical stages. Therefore, an approach is needed that not only integrates CNNs with additional features but is also specifically designed to extract clinically relevant texture characteristics and improve model robustness under imbalanced data conditions.

Based on this gap, this study proposes a Robust Intelligent CNN Model (RICNN) that integrates Gabor-based feature extraction with CNNs to enhance the detection of subtle microvascular patterns. Unlike previous hybrid approaches, the proposed method emphasizes texture-oriented feature integration aligned with the clinical characteristics of DR. In addition, this study incorporates a data-balancing strategy using SMOTE and training optimization with the Adam optimizer to improve model stability and convergence.

We explicitly evaluate the proposed model against Local Binary Pattern (LBP) and Color Histogram baselines on the Messidor dataset to validate its superiority in identifying clinically critical stages of DR.

This paper makes the following key contributions to the field of intelligence-based medicine:

1. Development of the RICNN: A hybrid architecture that synergizes Gabor-based texture enhancement with CNNs to improve the detection of subtle microvascular lesions in multi-grade DR.
2. Robust Methodology: The robustness of the proposed framework lies in its systematic pipeline, including standardized preprocessing, multi-scale Gabor feature extraction, fixed-length feature aggregation, feature-space balancing, and normalized CNN-based classification, which together support stable and consistent diabetic retinopathy grading.
3. The systematic application of SMOTE balancing and Adam optimization to resolve data disparity and ensure efficient convergence, guaranteeing reliable detection of rare but critical Proliferative DR cases.
4. Comprehensive Validation: A rigorous evaluation demonstrating that the RICNN outperforms LBP and Color Histogram baselines, offering a reliable, intelligent foundation for automated clinical screening.

The following sections are presented: Section 2 covers related work on DR classification. Section 3 details the materials, the proposed RICNN architecture, and the robust methodology. Section 4 discusses experimental results and clinical evaluation. Finally, Section 5 presents the conclusion and future research directions.

## 2. Related Works

This section reviews the evolution of diabetic retinopathy (DR) classification, shifting from pure deep learning architectures to hybrid feature-extraction strategies, and identifies the specific research gaps this study addresses.

### 2.1. Deep Learning in DR Detection and Its Limitations

Several recent studies have focused specifically on detecting diabetic retinopathy (DR) using deep learning techniques. The authors of ref. [30] introduced the Diabetic Retinopathy Compact Convolutional Transformer (DRCCT) model, which combines convolution and transformer techniques to improve retinal image classification. The authors of ref. [31] proposed a deep learning-based automatic classification method combined with novel preprocessing techniques and the use of ultra-wide-field (UWF) fundus image datasets to improve the accuracy of DR assessment. Meanwhile, ref. [32] developed the BathNet model, a deep learning-based approach that combines a Convolutional Neural Network (CNN) and a Transformer to more effectively detect small features such as microaneurysms, exudates, and hemorrhages.

Furthermore, ref. [17] proposed a genetic algorithm-based approach to optimize CNN architectures, using CNNs for feature extraction and support vector machines (SVMs) for classification. The authors of ref. [33] introduced the AC-DenseNet neural network architecture to improve DR classification performance by leveraging deeper connectivity. Furthermore, ref. [34] proposed a CNN model for the automatic detection of DR by integrating multi-view fundus images to enhance the model’s ability to capture retinal feature variations.

Although these various approaches have shown significant performance improvements, most research still focuses on a single aspect, such as image quality improvement, model optimization, or network architecture design. Therefore, an approach that integrates these strategies is needed to produce a more robust and accurate DR detection system.

### 2.2. Hybrid Approaches in Medical Computer Vision

To overcome the limitations of pure CNNs, recent research has proposed hybrid approaches that fuse automatic feature extraction with handcrafted domain knowledge.

- Gabor Filters: In broader domains, integrating CNNs with Gabor filters has been effective for distortion correction in geophysical data and texture enhancement in object detection [35,36]. However, these approaches are generally applied in a generic manner and are not specifically optimized for capturing clinically relevant retinal microvascular patterns.
- Local Binary Pattern (LBP): Approaches combining CNN + LBP serve as lightweight descriptors robust to lighting changes, helping CNNs recognize subtle texture patterns relevant to classification targets [37,38,39]. Nevertheless, LBP primarily captures local binary texture information and may fail to represent complex frequency-based vascular structures in DR images.
- Color Histograms: Integrating CNNs with Color Histograms provides global color representation, which has proven useful in distinguishing lesion types in skin cancer classification based on spectral differences [29]. However, such approaches often lack spatial and structural sensitivity, which limits their effectiveness in detecting fine-grained retinal abnormalities.

These studies collectively demonstrate that combining manual and automatic features enhances model generalization. However, most existing hybrid approaches rely on generic feature fusion strategies and do not explicitly consider task-specific feature design tailored to the clinical characteristics of DR.

### 2.3. Feature Extraction Specifically for Diabetic Retinopathy

Within the specific domain of DR, handcrafted features have shown promise in highlighting pathological biomarkers. The authors of refs. [11,12] utilized Gabor filters to highlight small blood vessel structures and lesions, effectively reducing noise and enhancing the CNN’s ability to recognize vascular patterns. Similarly, refs. [40,41,42] employed LBP to extract retinal micro-textures, which are effective for capturing local patterns but remain limited in representing high-frequency directional features associated with microvascular abnormalities. However, there is still limited work that systematically integrates multiple complementary feature types (e.g., texture, pattern, and color) within a unified CNN framework for DR classification.

Moreover, existing studies typically evaluate these features independently rather than investigating their combined effect in improving multi-stage DR classification performance.

### 2.4. Research Gap and Contribution

Despite these advancements, a critical gap remains. Most existing hybrid studies rely on standard convolutional architectures without identifying the optimal integration of retina-specific features. Previous works tend to focus on a single feature type (e.g., only Gabor or only LBP) separately and have not systematically compared the impact of different feature combinations—texture (Gabor), morphology/pattern (LBP), and color (Histogram)—on CNN performance specifically for multi-grade DR classification (four severity stages).

Furthermore, many existing hybrid approaches rely on generic feature fusion strategies, lacking task-specific integration mechanisms that align feature extraction with clinically relevant DR characteristics. This limitation underscores the need for a more structured, domain-aware integration framework.

This study aims to fill this gap by combining multiple handcrafted features with CNNs and by designing a task-specific integration strategy within a Robust Intelligent CNN (RICNN) framework. The proposed method systematically evaluates and integrates Gabor, LBP, and color-based features to enhance the detection of clinically significant DR stages. In addition, the model incorporates data balancing and optimized training to improve robustness across imbalanced multi-class scenarios, leading to more accurate and generalizable DR classification.

## 3. Materials and Methods

**Figure 1:**
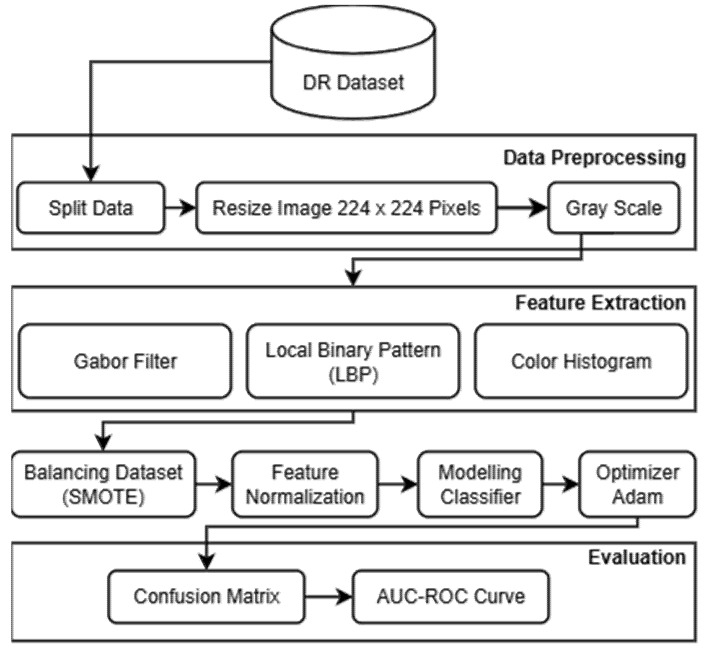
Research workflow.

The proposed research framework for developing a Robust Intelligent CNN Model (RICNN) is illustrated in Figure 1. The methodology is structured into several sequential stages, namely: data collection, feature extraction using three different approaches (Gabor filter, Local Binary Pattern, and color histogram), data balancing using SMOTE, feature normalization, CNN-based classification with Adam optimization, and performance evaluation. In this study, three parallel models are developed to systematically compare the effectiveness of different feature extraction techniques for diabetic retinopathy classification.

Each model follows an identical processing pipeline to ensure a fair comparison. The pipeline consists of feature extraction, SMOTE-based data balancing, standard scaling for feature normalization, CNN-based classification, and evaluation using multiple performance metrics. The main difference between the models lies in the feature extraction stage: Model 1 uses Gabor filters, Model 2 uses Local Binary Pattern (LBP), and Model 3 uses Color Histogram features. This design ensures a fair and consistent comparison across all models.

### 3.1. Dataset and Data Preprocessing

**Figure 2:**
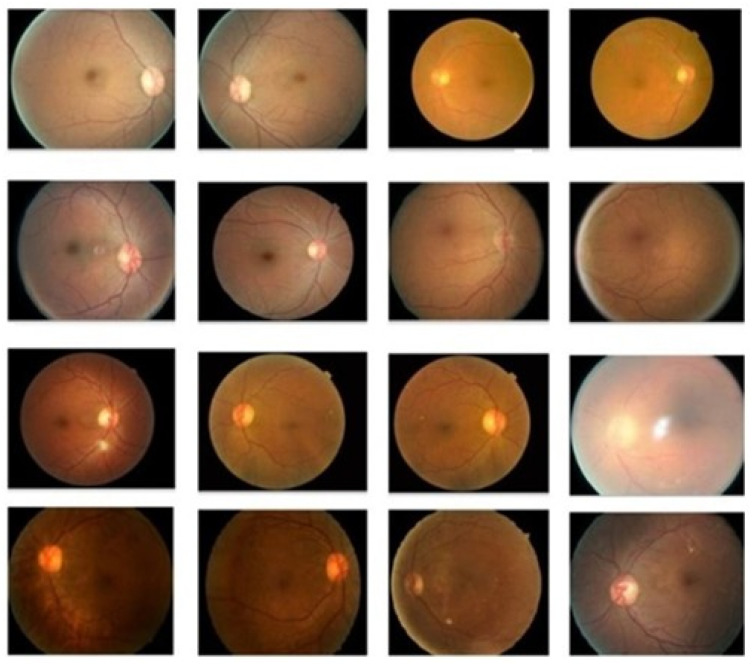
Fundus image of diabetic retinopathy.

The experimental validation was conducted using the Messidor dataset, which is a widely recognized benchmark for diabetic retinopathy classification [43]. The mapping between the Messidor grades and clinical categories is defined as follows: grade 0 corresponds to Mild NPDR (*n* = 546), grade 1 to Moderate NPDR (*n* = 254), grade 2 to Severe NPDR (*n* = 247), and grade 3 to Proliferative Diabetic Retinopathy (PDR) (*n* = 153). This mapping is adopted to ensure consistency with commonly used clinical interpretations in diabetic retinopathy classification studies. Representative samples of the fundus images are presented in Figure 2.

To ensure computational efficiency and compatibility with the CNN architecture, the dataset is randomly divided into training (80%), testing (10%), and validation (10%) sets. The splitting process is performed using a stratified sampling strategy to preserve the class distribution across all subsets, ensuring a fair and unbiased evaluation [44]. All images are resized from their original resolution (800 × 800 pixels) to 224 × 224 pixels to match the input requirements of the CNN model [45]. Furthermore, grayscale conversion is applied to the texture-based models (Gabor and LBP) to reduce computational complexity while preserving essential structural information, particularly retinal vascular patterns relevant to diabetic retinopathy detection [46]. For the Color Histogram model, the original RGB images are retained to preserve chromatic information required for feature extraction. The pixel intensity values are normalized to the range [0, 1] to stabilize the training process and improve convergence.

### 3.2. Feature Extraction Framework

Feature extraction is a critical component of the proposed intelligent system, transforming raw pixels into meaningful clinical descriptors. This study implements and systematically compares three feature extraction strategies, with a primary focus on Gabor-based feature extraction as a task-specific enhancement designed to improve the representation of microvascular patterns in diabetic retinopathy (DR) images within the RICNN framework. Each feature extraction method is applied independently to construct three separate models, enabling a fair and structured comparative analysis.

#### 3.2.1. Gabor Filter (Proposed Intelligent Enhancement)

Gabor feature extraction is performed as an offline preprocessing step before model training and evaluation. This design ensures that texture enhancement is achieved without increasing computational complexity during inference, as the classification stage is handled entirely by the forward propagation of the proposed RICNN architecture. Unlike conventional hybrid approaches that integrate handcrafted features through direct fusion during training, the proposed method applies Gabor filtering as a structured preprocessing step, allowing the CNN to learn from texture-enhanced inputs without modifying its internal architecture.

The Gabor filter serves as the primary feature enhancement mechanism in the proposed framework and is selected for its ability to mimic the texture-discrimination properties of the human visual system. This property makes Gabor filters particularly effective for modeling directional and frequency-sensitive patterns associated with retinal microvascular structures. The process begins by converting the fundus image to grayscale, ensuring computational efficiency while preserving essential structural information. The Gabor kernel is convolved with the input image to generate feature maps that highlight specific orientations and frequencies. This process enhances clinically relevant features such as blood vessels, microaneurysms, and hemorrhages, which are critical for accurate multi-grade DR classification [10,12,35,47,48,49]. By emphasizing high-frequency microvascular patterns prior to CNN processing, the proposed approach enables the model to learn more discriminative representations compared to conventional CNN models that rely solely on raw pixel inputs. The following equation can define a Gabor filter:

(1) $$g\;(x,y;\;\lambda ,\theta ,\psi ,\sigma ,\gamma )=\exp\left(-\frac{x^{\prime 2}+\gamma^{2}y^{\prime 2}}{{2\sigma }^{2}}\right)\cos\left(2\pi \frac{x^{\prime }}{\lambda }+\psi \right)\;\;where,\;x^{\prime }=x\cos\theta +y\;sin\;\theta {\;,\;y}^{\prime }=-x\sin\theta +y\;cos\;\theta$$

where x and y denote the spatial coordinates (horizontal and vertical) of a pixel, λ represents the wavelength, θ defines the orientation of the Gabor filter, and σ is the standard deviation of the Gaussian envelope. ψ represents the phase offset and γ denotes the spatial aspect ratio. In this study, the Gabor filter parameters are defined as follows: λ=4.8,ψ=0,σ=2,and γ=0.5. These values are selected empirically to capture multi-scale and orientation-sensitive retinal texture patterns effectively. The filter bank is configured with frequencies: {2, 1.9, 1.7, 1.5, 1.3, 1, 0.9, 0.7, 0.5} and theta orientations: $\{0^{o},\;30^{o},\;45^{o},60^{o},\;90^{o}\}.$ All combinations of these frequencies and orientations are systematically applied to each retinal image, generating multiple Gabor-filtered feature maps. The resulting feature maps are processed in the feature extraction stage, where each filtered output is flattened into a feature vector, and all feature vectors are concatenated to form a single high-dimensional feature representation. Each retinal image is resized to 224 × 224 pixels before filtering. This feature representation is subsequently normalized and used as input to the classification framework after data balancing.

#### 3.2.2. Comparative Baselines: LBP and Color Histogram

To rigorously validate the effectiveness of the proposed Gabor-based approach, two established feature extraction methods are implemented as comparative baselines:

Local Binary Pattern (LBP): LBP is used to capture local micro-texture patterns. It works by comparing a central pixel with its neighboring pixels, generating a binary code that describes the local contrast. To extract LBP features, an operator is applied to each pixel in a segment. In the basic mode, the brightness value of each central pixel (g(c)) is compared with the brightness value of its neighboring pixels (g(p)) within a certain neighborhood (e.g., P neighboring pixels within a radius R). If the brightness intensity of a neighboring pixel is greater than or equal to that of the central pixel, then the pixel is assigned a value of 1; otherwise, it is assigned a value of 0. These binary values are arranged in sequence and form a binary number, which is considered the LBP code for the central pixel. In fundus images, LBP helps distinguish between smooth normal tissue and irregular pathological spots. Although LBP is robust to illumination changes, it lacks the directional and frequency sensitivity provided by Gabor filters [13,50,51,52,53,54]. The following equation expresses LBP:

(2) $$LBP\;\left(P,R\right)=\sum_{p=0}^{p=1}\left[s\left(g\left(p\right)-g\left(c\right)\right)x2^{p}\right]$$

where P (number of neighbors) denotes the number of neighboring pixels considered around the center pixel. In this study, (P = 8) is used. R (Radius) represents the distance between the center pixel and its neighboring pixels. The final configuration R=1. $g\left(c\right)$ denotes the gray level value of the center pixel, while $g\left(p\right)$ represents the gray-level value of the p-th-neighbor pixel. $s\left(x\right)$ is the threshold function. $2^{p}$ represents the binomial weight assigned to each neighbor to convert the binary pattern into a decimal value. The resulting LBP codes are summarized using a histogram with 10 bins, forming a compact texture feature vector. Although multiple radius values ((R=1) to (5)) were explored during a parameter tuning stage, the final configuration ((P = 8), (R=1)) was selected based on validation performance.

Color Histogram: This method analyzes the image’s chromatic distribution. Unlike the texture-based methods (Gabor and LBP), the Color Histogram extracts feature vectors from the Red, Green, and Blue (RGB) channels. This representation is relevant for DR analysis, as specific color distributions are associated with pathological features such as hemorrhages and exudates [29]. In general, histograms are often normalized by dividing each gray level value by the total number of pixels in the image, represented by the letter n.

(3) $$\left(rk\right)=\frac{nk}{n}$$

where rk is the normalized histogram value at intensity level k, nk is the number of pixels with intensity level k, and n is the total number of pixels in the image.

In the experiment, color information was extracted using a histogram representation in the RGB color space. Each color channel was quantized into 32 bins to describe the distribution of pixel intensities. The histograms of the Red, Green, and Blue channels were concatenated to form a 96-dimensional color feature vector, serving as the basic color descriptor in the classification framework. The feature extraction method and feature dimensions are presented in Table 1.

### 3.3. Flowchart of Each Model

**Figure 3:**
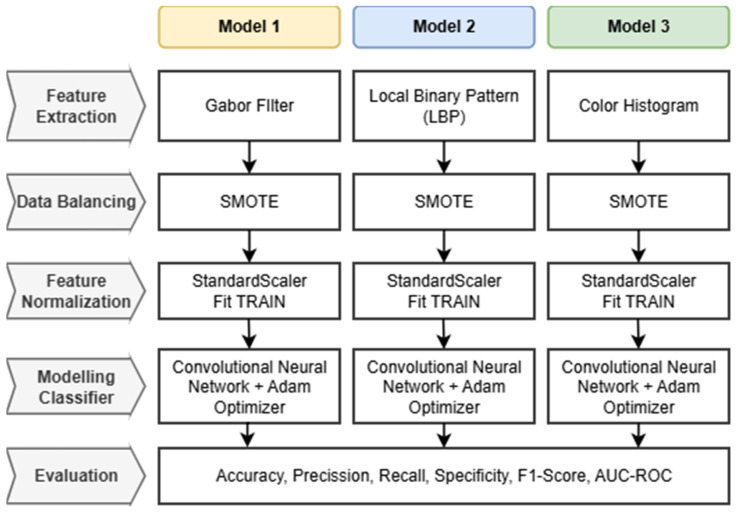
Workflow of the proposed multi-model classification framework.

This subsection describes the integration of the proposed models within a unified workflow. As shown in Figure 3, the flowchart summarizes the processing pipeline, consisting of five key stages: feature extraction, data balancing, feature normalization, modeling classification, and evaluation. The workflow is designed to ensure a structured and task-specific integration of handcrafted features with CNN-based learning.

The pipeline begins with preprocessed fundus images serving as input. Depending on the model scheme, the images undergo distinct feature extraction processes: Gabor filtering for texture orientation, LBP for local pattern contrast, or the Color Histogram for chromatic distribution. To handle class imbalance, the extracted features are processed using the Synthetic Minority Over-sampling Technique (SMOTE). In contrast to conventional hybrid approaches that apply simple feature concatenation, the proposed framework processes each feature type through a standardized transformation pipeline, using a StandardScaler (Fit TRAIN) to preserve discriminative characteristics while ensuring compatibility with CNN input representations. Finally, these processed feature vectors are fed into the Convolutional Neural Network (CNN) classifier integrated with the Adam Optimizer, where the network learns hierarchical representations guided by the pre-extracted domain-specific features. This setup enables more accurate multi-grade classification into Mild, Moderate, Severe, or Proliferative DR classes.

This structured pipeline allows the model to systematically compare and leverage multiple feature extraction strategies, highlighting the effectiveness of Gabor-based enhancement in capturing clinically relevant microvascular patterns compared to other feature types.

### 3.4. Data Balancing

A critical challenge in this dataset is the significant class imbalance, where Severe cases are underrepresented. To build a robust model that does not bias toward the majority class (Mild), we implemented the SMOTE (Synthetic Minority Oversampling Technique) [55]. SMOTE is configured with (k = 5) nearest neighbors and applied exclusively to the training set after data splitting. The synthetic samples are generated in the feature space after feature extraction, where all features are normalized prior to oversampling. SMOTE synthesizes new minority instances by interpolating between existing samples, ensuring a balanced distribution across all four classes. Although applied in a high-dimensional feature space, the combination of normalization and structured feature extraction ensures that the generated samples remain meaningful and discriminative.

**Figure 4:**
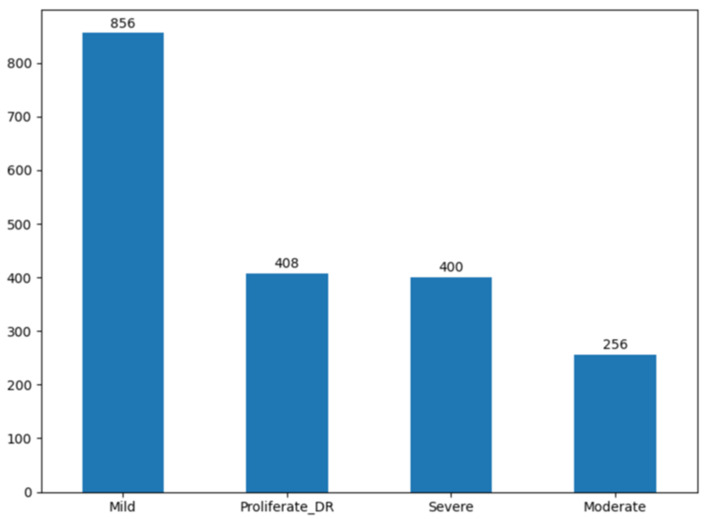
Distribution of the dataset before SMOTE.

**Figure 5:**
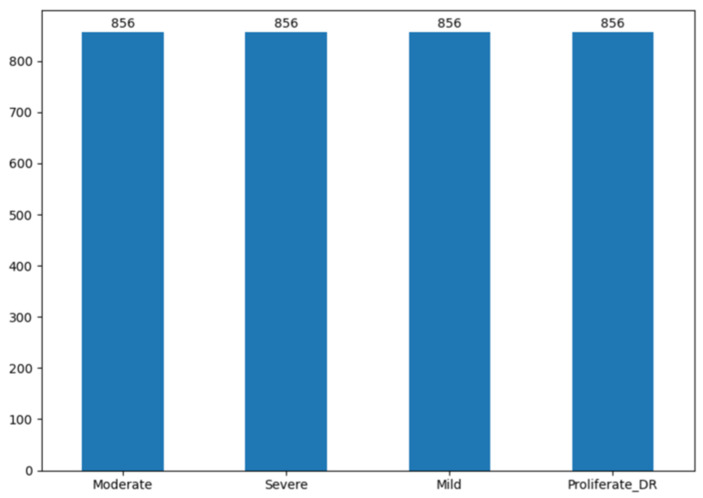
Distribution of the dataset after SMOTE.

The distributions of the dataset before and after SMOTE are shown in Figure 4 and Figure 5, respectively. The test set is kept unchanged and reflects the original class distribution. Therefore, the confusion matrices report results on an imbalanced test set, ensuring a realistic evaluation scenario without data leakage.

SMOTE balancing was applied exclusively to the training set after data splitting. The test set was kept unchanged and reflects the original class distribution. Therefore, the confusion matrices report results on an imbalanced test set.

### 3.5. Feature Normalization

To ensure model stability and efficient convergence, Feature Normalization was performed using the StandardScaler technique. As explicitly illustrated in the workflow (Figure 3), this process adheres to a strict “Fit on Training” strategy. This step transforms the feature vectors, whether derived from the proposed Gabor-based enhancement or the comparative baselines (LBP and Color Histogram), into a standard normal distribution with a mean of 0 and a standard deviation of 1. Crucially, the scaling parameters were computed solely from the training dataset and subsequently applied to the validation and test sets. This methodology prevents data leakage, ensuring that the model’s evaluation remains unbiased, while simultaneously preventing variables with larger magnitudes from dominating the learning process and accelerating optimization [56].

### 3.6. Modeling Classifier and Optimizer (RICNN Architecture and Optimization)

Classification systems aid in the differential diagnosis of underlying pathologies. The classification model proposed in this study uses a Robust Intelligent CNN (RICNN) model. The core of the proposed RICNN is a deep learning architecture explicitly designed to process texture-enhanced feature maps [57]. The network leverages local receptive fields and weight sharing to ensure high sensitivity to local pathological patterns (such as microaneurysms) while maintaining translation invariance. The architecture comprises sequential convolutional layers for hierarchical feature extraction, Rectified Linear Unit (ReLU) activation functions to introduce non-linearity, and pooling layers to provide partial invariance to shifts and scale deviations [46]. The technical specifications of the RICNN architecture are shown in Table 2.

Specifically, the model consists of two convolution layers with 32 and 64 filters, respectively, using a kernel size of 3 × 3 and a stride of 1. Each convolution layer is followed by a Batch Normalization layer, a 2D Max Pooling layer with a pool size of 2 × 2, and a Dropout layer to prevent overfitting. The feature maps are then flattened into a 186,624-dimensional vector before being passed to a Dense layer with 128 neurons. The final output size is 4 neurons with a Softmax activation function for multi-level classification.

To ensure efficient convergence and model stability, the RICNN is trained end-to-end using the Adam (Adaptive Moment Estimation) Optimizer. Adam was specifically selected for its ability to adapt learning rates for individual parameters based on first- and second-moment estimates, which is critical for optimizing complex hybrid features where standard SGD might struggle [58,59,60]. The model’s primary hyperparameters (Adam Optimizer) in the experiments have technical significance: learning rate 0.0005, beta 1 (β_1_) 0.9, beta 2 (β_2_) 0.999, and epsilon (ε) 1 × 10^−7^.

Furthermore, to enhance generalization and prevent overfitting on the imbalanced medical dataset, the training pipeline rigorously incorporates Batch Normalization after convolution operations, Dropout (0.5) within the fully connected layers, and a dynamic learning rate schedule based on validation performance [61]. For comparison purposes, the same CNN architecture is also trained directly on raw retinal images without employing any feature extraction techniques.

### 3.7. Performance Evaluation

To rigorously assess the diagnostic capabilities of the proposed RICNN and comparative models, two primary evaluation protocols were established: the confusion matrix and the Receiver Operating Characteristic (ROC) curve.

#### 3.7.1. Confusion Matrix and Clinical Metrics

The confusion matrix serves as the foundational tool for evaluating the classification model, mapping the predicted labels against the ground truth. From the values of True Positive (TP), True Negative (TN), False Positive (FP), and False Negative (FN), we derived four key clinical metrics to provide a comprehensive performance overview:

- Accuracy: Measures the overall correctness of the model across all grades.
- Precision: Indicates the reliability of positive predictions, crucial for minimizing false alarms.
- Recall (Sensitivity): Measures the model’s ability to correctly identify positive cases. In the context of DR screening, recall is prioritized to minimize False Negatives (missed diagnoses), which could lead to irreversible blindness if untreated.
- F1-Score: The harmonic mean of precision and recall, providing a balanced metric for cases with uneven class distributions.

#### 3.7.2. Area Under the ROC Curve (AUC-ROC)

To evaluate the model’s discriminative ability across different decision thresholds, we utilized the Area Under the Receiver Operating Characteristic (AUC-ROC) curve. The ROC represents the trade-off between the True Positive Rate (Sensitivity) and the False Positive Rate (1-specificity). The AUC quantifies the degree of separability between classes; a higher AUC (approaching 1.0) indicates the model’s superior ability to distinguish between patients with varying degrees of diabetic retinopathy severity (e.g., distinguishing Moderate from Severe cases), which is essential for accurate triage.

## 4. Results and Discussion

### 4.1. Performance Evaluation of the Proposed RICNN (Gabor-Enhanced) (Model 1)

**Figure 6:**
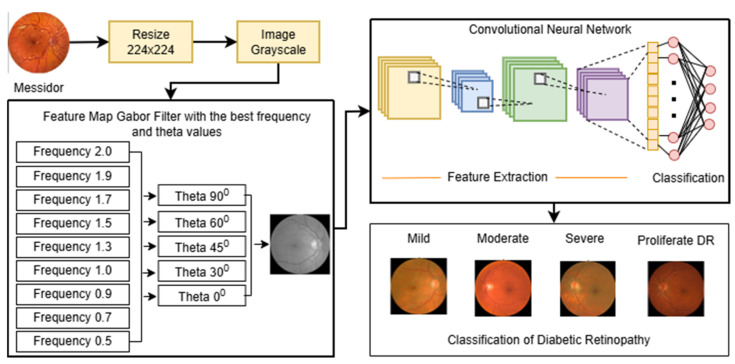
The Gabor filter integration experiment flow.

The primary objective of this experimental phase was to evaluate the diagnostic efficacy of the Robust Intelligent CNN Model (RICNN). The foundational component of this intelligent system is the integration of Gabor filters, chosen for their optimal localization properties in both spatial and frequency domains. Unlike standard preprocessing, the Gabor enhancement acts as a “texture-aware” filter, explicitly extracting fine retinal textures and suppressing residual noise to aid the CNN in detecting subtle pathologies [22]. The experimental flow for Gabor filter integration is shown in Figure 6.

The effectiveness of this feature enhancement is visually demonstrated in Figure 6. The figure illustrates how applying Gabor filters with varying frequencies (*f*) and orientations (θ) successfully highlights the complex vascular architecture—ranging from major trunks to fine capillaries—which provides the neural network with critical prior knowledge for classification.

**Figure 7:**
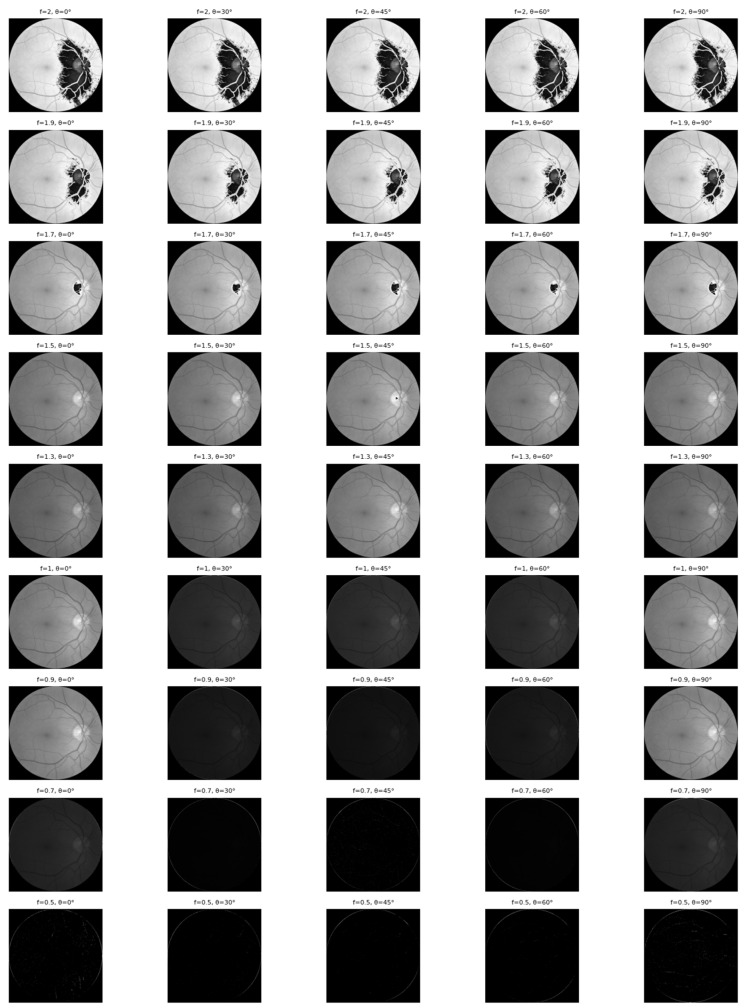
Results of applying a Gabor filter to a fundus image of diabetic retinopathy.

A visualization of the Gabor filter bank response is presented in Figure 7. This figure illustrates the effectiveness of combining Gabor filters with varying frequencies and orientations to comprehensively extract retinal blood vessel features. Relying on a single orientation would limit visibility to vessels aligned in only one specific direction; however, by employing multiple orientations, the model captures vascular structures across all trajectories. Complementing this, the frequency (f) determines the size of the sine wave within the Gabor kernel, thereby governing the granularity of the captured texture.

A high *f*-value facilitates the detection of fine details, such as small capillaries and micro-textures, whereas a small *f*-value is more sensitive to coarse structures like main vascular trunks. Simultaneously, the orientation parameter (θ, theta) set at 0°, 30°, 45°, 60°, and 90° controls the directionality of feature highlighting. Specifically, θ = 0° enhances horizontal vessels with high contrast, θ = 90° emphasizes vertical structures, and intermediate angles (30°, 45°, 60°) effectively highlight oblique or diagonal vascular paths [12]. Through a series of experimental evaluations, it was determined that the configuration of *f* = 9 and θ = 30° provided the most significant contribution to the model’s discriminative power, ultimately achieving an overall classification accuracy of 89%. Collectively, this multi-scale and multi-orientation approach produces feature maps that vividly highlight the entire retinal vascular network, providing the CNN with robust prior knowledge.

**Figure 8:**
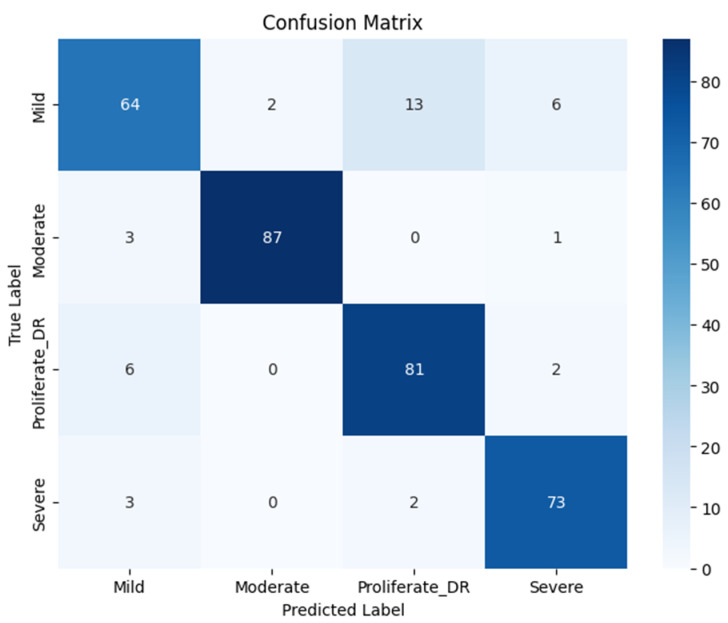
Confusion matrix for diabetic retinopathy classification using Gabor features and a CNN-based classifier, evaluated on the original test set without SMOTE balancing.

Following the feature enhancement process, rigorous data preparation was executed. The feature vectors were normalized using a StandardScaler, with the scaling parameters fitted solely to the training data and then applied to the test set to maintain experimental validity. Subsequently, the RICNN classifier was trained using the Adam Optimizer to facilitate efficient weight convergence. The comprehensive classification performance is detailed in Table 3 and visualized in Figure 8 through the confusion matrix.

The quantitative evaluation demonstrates the robustness of the proposed model. As shown in Table 3, the RICNN achieved an overall accuracy of 89%, indicating high reliability in distinguishing between the four severity grades of diabetic retinopathy. Beyond accuracy, the model exhibited a strong balance between sensitivity and specificity, recording an average precision of 88.75%, a recall of 89%, and an F1-score of 89%. Notably, both the macro-average and weighted average for these metrics aligned at 89%, confirming that the SMOTE balancing technique successfully mitigated the bias typically found in imbalanced medical datasets. This consistency suggests that the RICNN (Gabor-Enhanced) does not favor the majority class but recognizes all categories—from Mild NPDR to Proliferative DR—with equal effectiveness.

Analysis of class-wise performance metrics reveals clinically relevant trade-offs in sensitivity–specificity across different stages of diabetic retinopathy. The proposed model demonstrates high recall for clinically significant stages, particularly for Moderate (96%) and Severe (94%) cases, indicating a strong ability to correctly identify patients who require medical attention. This characteristic is especially important in screening scenarios, where minimizing False Negatives is critical to prevent delayed diagnosis and disease progression.

While the recall for Mild cases is comparatively lower (75%), this reflects the inherent visual subtlety of early-stage manifestations and represents a common challenge in automated diabetic retinopathy analysis. Nevertheless, the overall balanced performance, as reflected by macro- and weighted-average F1-scores of 89%, suggests that the proposed framework maintains an effective balance between sensitivity and precision across severity levels. These findings support the potential clinical utility of the proposed model as a screening-oriented decision support tool.

**Figure 9:**
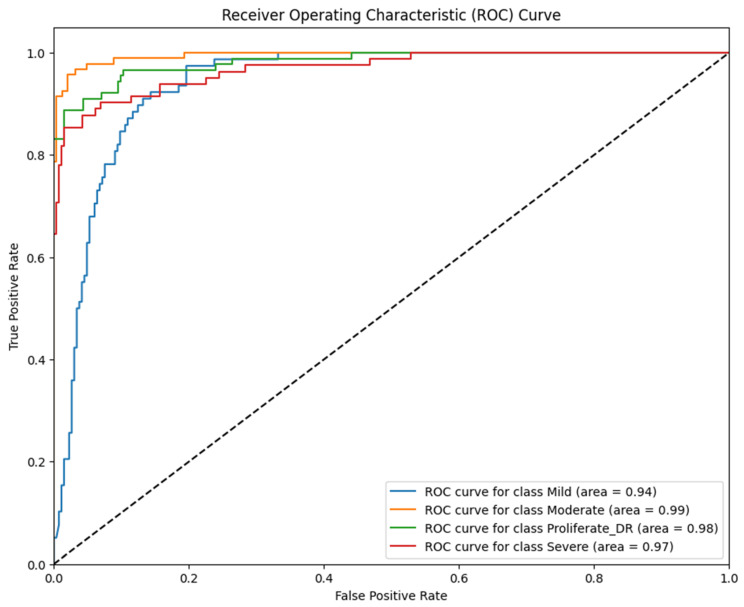
ROC curve of diabetic retinopathy fundus image classification using the Gabor filter method and CNN.

Complementing the discrete classification metrics presented in Table 2, the model’s discriminative ability was rigorously evaluated using the Receiver Operating Characteristic (ROC) curve analysis. This evaluation is critical in medical diagnostics as it illustrates the trade-off between the True Positive Rate (Sensitivity) and the False Positive Rate (1-specificity) across all possible decision thresholds. By analyzing the Area Under the Curve (AUC), we can quantify the model’s robustness in distinguishing between adjacent severity grades (e.g., separating Moderate from Severe cases), which is essential for accurate patient triaging. The resulting ROC curves for the proposed RICNN, which visualize this diagnostic performance across the four severity classes, are depicted in Figure 9.

The ROC curves depicted in Figure 8 quantitatively validate RICNN’s superior discriminatory ability across all severity levels. Unlike baseline classifiers that often struggle with fine boundaries between progressive stages, RICNN achieved near-perfect separation. Specifically, the model recorded AUC scores of 94% for Mild, 99% for Moderate, 97% for Severe, and 98% for Proliferative DR.

These results are clinically significant, as the high AUC values for the advanced stages (98% and 97%) indicate that the model is robust in distinguishing critical cases requiring immediate medical attention. This confirms that the integration of Gabor-based texture features effectively widens the decision margin between classes, ensuring an accurate and reliable basis for automated diagnostic systems in real-world clinical settings.

From a computational standpoint, the proposed RICNN architecture comprises approximately 23.9 million trainable parameters, resulting in a model size of around 91 MB. This reflects a moderate memory requirement that can be accommodated by typical clinical computing systems. As runtime performance analysis was beyond the scope of this work, inference latency was not quantitatively evaluated.

### 4.2. Comparative Analysis with Baselines

To rigorously validate the superiority of the proposed RICNN, this study conducted a detailed benchmarking against two established feature extraction methods: the Local Binary Pattern (LBP) and Color Histogram. To ensure a fair and consistent comparison, both baseline models strictly adhered to the identical four-stage workflow illustrated in Figure 3. This includes the application of Standard Scaler normalization, fitted exclusively on the training data to prevent data leakage, followed by training the CNN classifier using the Adam Optimizer with hyperparameters identical to those of the primary RICNN framework.

#### 4.2.1. Performance Evaluation of LBP-CNN (Model 2)

**Figure 10:**
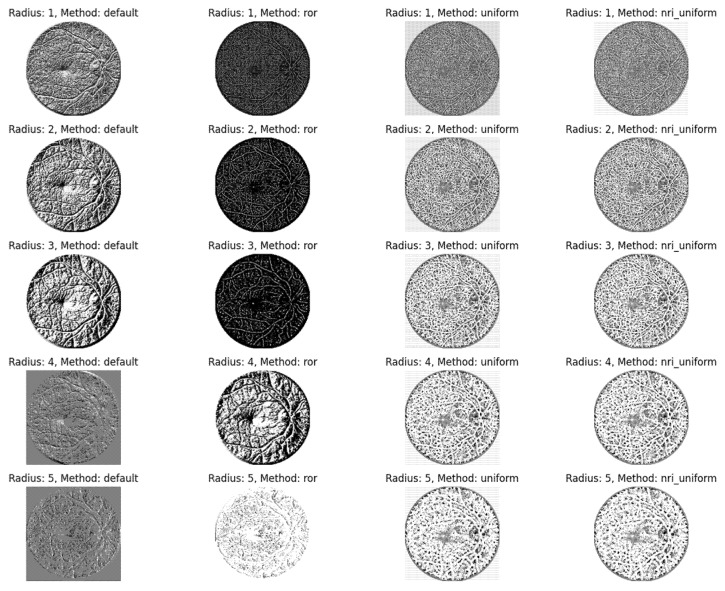
Results of applying LBP to the fundus image of diabetic retinopathy.

The second experimental phase evaluated the performance of the CNN model integrated with Local Binary Pattern (LBP). LBP was employed as a texture descriptor to encode local contrast by comparing the intensity value of a central pixel with its circular neighborhood. To optimize feature extraction, a parameter-tuning stage was conducted using various radius values (ranging from 1 to 5) and different LBP configurations, including the default, rotation-invariant (*ror*), uniform, and non-rotation-invariant uniform (*nri_uniform*) patterns. Based on validation performance, the final configuration was selected as (P = 8), (R = 1), using the uniform LBP method with a 10-bin histogram representation. This process generates a binary representation that effectively captures the micro-texture of the retina. The visual output of these LBP transformations is presented in Figure 10.

**Figure 11:**
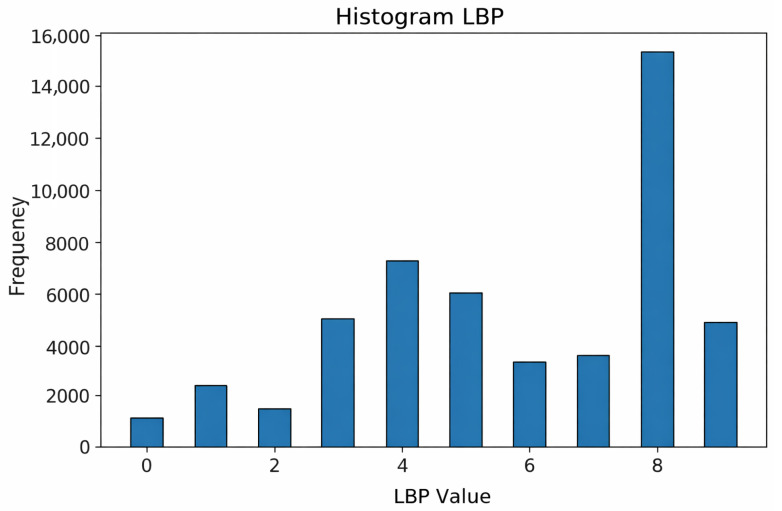
Histogram of LBP in the fundus image of diabetic retinopathy.

The resulting LBP images are quantified into feature vectors representing the distribution of texture patterns, as visualized in the LBP Histogram (Figure 11). The histogram consists of representative bins indicating the frequency of detected patterns.

A detailed analysis of Figure 11 reveals that the most dominant feature value is 8. This peak indicates the high prevalence of texture patterns where neighbors are brighter than the center pixel, which typically corresponds to high-contrast areas such as blood vessel boundaries and bright lesions (exudates). Other values, such as 4, also appear frequently, representing moderate edges, while low values (0 to 2) are relatively rare. These statistical features confirm that the fundus images contain complex texture information that serves as the input for the CNN classifier.

**Figure 12:**
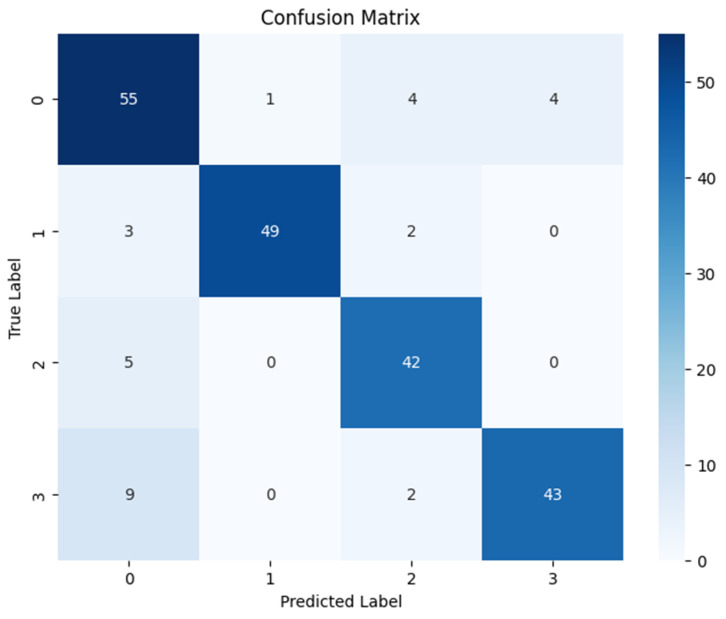
Confusion matrix for diabetic retinopathy classification using LBP features and a CNN-based classifier, evaluated on the same original test set.

Quantitative Performance of LBP-CNN: Following the training phase, the LBP-CNN model was evaluated on the test set. The comprehensive performance metrics are detailed in Table 4, and the confusion matrix is presented in Figure 12.

The results indicate that the LBP-CNN achieved an overall accuracy of 86%, with a precision of 87.25%, a recall of 86.50%, and an F1-score of 86.75%. While these scores demonstrate good classification capability, they are notably lower than the proposed RICNN (89%). The confusion matrix reveals a specific limitation: while the model performed reliably for the Moderate class (recall 91%), it showed reduced sensitivity for the Proliferative DR class (recall 80%), misclassifying several critical advanced cases as lower severity grades. This suggests that while LBP is robust for detecting localized lesions, it lacks the directional sensitivity required to fully trace the complex vascular proliferation characteristic of advanced DR.

**Figure 13:**
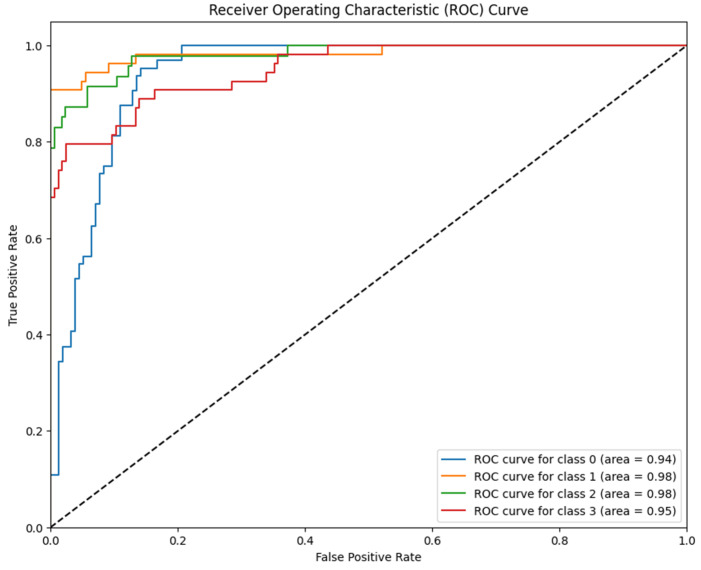
ROC curve of diabetic retinopathy fundus image classification using LBP and CNN methods.

Discriminative Ability (LBP-CNN): The discriminative power of Model 2 is further illustrated by the ROC curves in Figure 13.

The model maintained robust performance with AUC values ranging from 94% to 98% across the four classes. Specifically, the Moderate and Severe classes achieved an AUC of 98%, followed by Proliferative DR at 95% and Mild at 94%. Although these values indicate strong probabilistic classification capabilities, the discriminative power for the Proliferative stage (95%) was notably lower than that of the RICNN (99%), confirming that the lack of global directional filtering impacts the detection of advanced vascular anomalies.

#### 4.2.2. Performance Evaluation Color Histogram-CNN (Model 3)

The third experimental phase assessed the efficacy of spectral features using the Color Histogram method. Unlike the texture-based approaches (Gabor and LBP), this method explicitly analyzes the chromatic distribution of the retina across the Red, Green, and Blue (RGB) channels. During the parameter tuning stage, different bin sizes (e.g., 8 bins per channel) were evaluated to analyze their impact on feature representation. The final configuration uses 32 bins per channel, resulting in a 96-dimensional feature vector, as described in the Methodology section. This configuration was selected based on validation performance, as it provides a more detailed representation of color distribution. This representation enables the model to capture color-specific biomarkers, such as the deep red of hemorrhages and the yellowish-white appearance of hard exudates.

**Figure 14:**
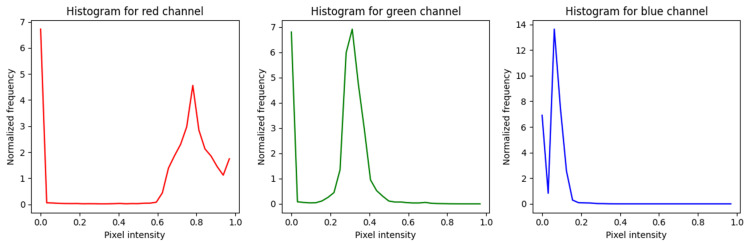
Results of applying a Color Histogram to a fundus image of diabetic retinopathy.

The resulting spectral distributions are visualized in Figure 14. A detailed analysis of the histograms reveals distinct characteristics for each channel.

The Red channel exhibits a broad intensity distribution with dual peaks, reflecting substantial variation in vascularization and hemorrhage, which are essential for DR diagnosis. The Green channel shows a more concentrated intensity profile, often used to contrast vessels against the fundus background. Conversely, the Blue channel displays a sharp peak at low intensities, confirming that blue spectral information is less dominant in retinal imaging. These color signatures serve as the primary input for the CNN classifier.

**Figure 15:**
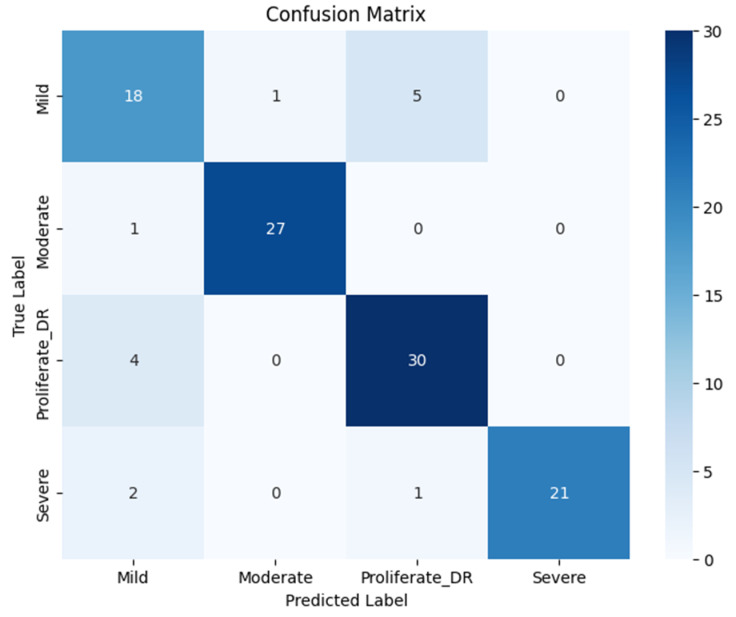
Confusion matrix classification of diabetic retinopathy fundus images using the Color Histogram and CNN methods.

Upon evaluating the model on the independent test dataset, the quantitative results presented in Table 5 and the confusion matrix in Figure 15 indicate competitive performance. The Color Histogram-CNN achieved an overall accuracy of 87%, with a precision of 87.25%, a recall of 86.75%, and an F1-score of 87%.

A closer inspection of the class-wise performance reveals specific strengths and limitations of spectral analysis. The model demonstrated exceptional sensitivity for the Moderate NPDR class, achieving a recall of 96%. This suggests that color features are highly effective at detecting the distinct reddish hemorrhages and yellowish exudates typical of the Moderate stage. However, for the Proliferative DR class, the recall dropped to 88%, which is notably lower than the 91% achieved by the proposed RICNN. This decline indicates that while color histograms effectively flag discolored lesions, they lack the spatial and morphological context required to accurately trace the complex neovascularization (new abnormal blood vessel formation) that characterizes advanced proliferation.

**Figure 16:**
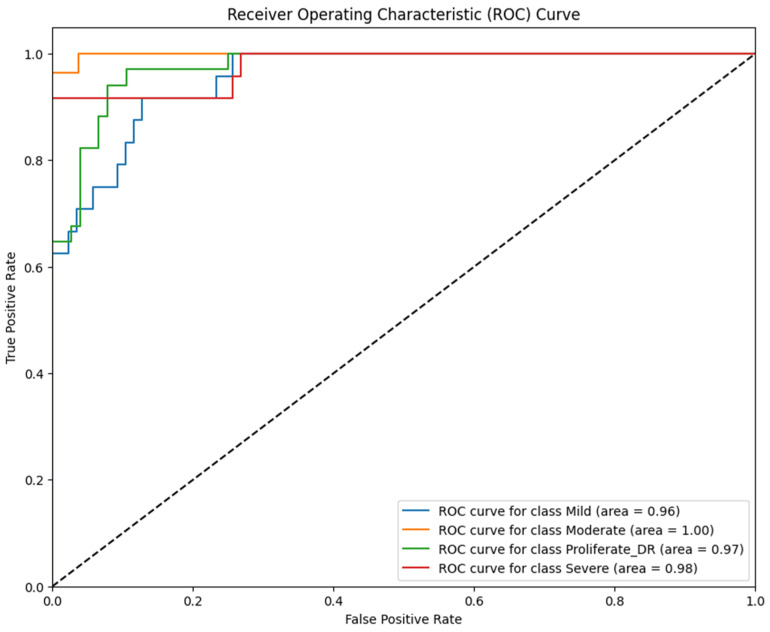
ROC curve of diabetic retinopathy fundus image classification using Color Histogram and CNN methods.

The discriminative capability of Model 3 is further elucidated by the ROC curves in Figure 16.

Based on Figure 16, the model exhibited excellent separability, particularly for the Moderate class, achieving a perfect AUC of 100%. This reinforces the finding that spectral distinctiveness is maximized at the Moderate stage, where color contrast is most vivid. The AUC values for other classes remained high—96% for Mild, 98% for Severe, and 97% for Proliferative DR. However, similar to the LBP model, the discriminative power for the most critical advanced stages did not exceed that of RICNN (99%), confirming that relying solely on color distribution without texture orientation limits the diagnostic ceiling for complex cases.

### 4.3. Discussion

The experimental results demonstrate that the proposed model achieves 89% accuracy in classifying fundus retinal images, effectively validating its robustness in detecting diabetic retinopathy (DR).

Compared with the existing literature, the proposed approach shows superior performance. Specifically, ref. [31] reported an accuracy of 66% using ResNet50, while the MVDRNet architecture utilized in ref. [34] achieved 77.75% using MVDRNet. In addition, ref. [62] obtained an accuracy of 84.6% using a Siamese-based Convolutional Neural Network, and ref. [63] reported 74% accuracy with the MFgcForest method. These comparisons clearly indicate that the proposed method surpasses several existing approaches by providing a more specialized feature extraction pipeline for medical imaging.

The observed improvement can be attributed to the synergistic integration of several key components. First, Gabor-based feature extraction significantly improves the representation of clinically relevant texture patterns in retinal images. Second, applying SMOTE mitigates the inherent class imbalance by augmenting minority-class representations, thereby ensuring a more balanced sensitivity across all DR grades. Third, leveraging the Adam optimizer contributes to more stable training and faster convergence. The combination of these components enables the model to achieve superior performance, particularly in capturing the nuances of multi-grade DR classification.

Nevertheless, despite these advancements, this study has several limitations. The model was evaluated on a specific dataset, which may affect its generalizability to unseen or more diverse data. Furthermore, variations in image quality and class imbalance remain challenging factors that may influence classification performance in real-world clinical settings.

For future work, it is recommended to utilize multi-center and more diverse datasets to improve model generalization. In addition, exploring attention-based architectures or vision transformers and integrating complementary techniques may further enhance the performance of DR detection systems.

#### 4.3.1. Comparative Analysis of Feature Extraction Strategies

This study aims to identify the most effective feature extraction strategy for the automatic classification of diabetic retinopathy (DR) severity. For comparison purposes, the performance of a baseline CNN trained directly on raw retinal images is also included. The summary of performance metrics for all evaluated models is presented in Table 6.

A comparative analysis of these results establishes a clear performance hierarchy: the proposed RICNN (Robust Intelligent CNN) or Model 1 (Gabor + CNN) achieved the highest overall efficacy with an accuracy of 89% and F1-score of 89%, followed by the Color Histogram-CNN (Model 3) at 87%, and the LBP-CNN (Model 2) at 86%. In contrast, the baseline CNN trained directly on raw retinal images achieved a substantially lower accuracy of 36%, highlighting the limited discriminative capability of raw image-based learning for multi-grade diabetic retinopathy classification under the same experimental settings, likely due to the high complexity and subtle spatial variations of DR lesions that require explicit feature enhancement.

Biological Justification for Gabor Superiority: The superiority of the RICNN (Model 1) can be directly attributed to the retina’s inherent biological characteristics. Diabetic retinopathy is fundamentally a vascular disease characterized by structural changes, including vessel dilation, tortuosity, and neovascularization. The Gabor filter, with its tunable orientation (θ) and frequency (*f*) parameters, acts as a “directional edge detector” mathematically analogous to the visual processing in the mammalian cortex. This allows the RICNN to explicitly capture the linear and directional nature of blood vessels. As evidenced in the ROC-AUC analysis (Table 7), Model 1 achieved exceptional discriminative power for the most critical advanced stages: Severe (97%) and Proliferative DR (99%). This confirms that texture-orientation features are crucial for identifying the chaotic vascular growth that defines advanced DR, a capability that standard texture or color features lack.

The results and how they can be interpreted in light of previous studies and the working hypotheses: The findings and their implications should be discussed in the broadest context possible. Future research directions may also be highlighted.

Analysis of Baseline Limitations: Model 3 (Color Histogram) demonstrated a unique strength in the Moderate NPDR class, achieving the highest AUC of 100% (Table 7). This is likely because the Moderate stage is clinically defined by distinct color-based lesions—red hemorrhages and yellow hard exudates—that are easily captured by spectral analysis. However, its discriminative power notably declined in the Severe and Proliferative stages (AUC 95–98%). This suggests that while spectral features effectively flag discolored lesions, they are insufficient for detecting structural anomalies (such as new transparent vessels) that lack strong color contrast against the background.

Similarly, Model 2 (LBP) provided consistent but lower performance (accuracy 86%). While LBP is effective for analyzing localized micro-textures, its typical rotation-invariant nature lacks the global directional sensitivity required to trace long vascular paths. Consequently, it struggled to distinguish the subtle transitions in vessel complexity between Severe NPDR and Proliferative DR, resulting in the lowest sensitivity for advanced cases. In contrast, the baseline CNN trained directly on raw retinal images exhibited the lowest AUC value among all evaluated models, further indicating its limited discriminative capability under the same experimental conditions.

Clinical Implications The comparison highlights a critical clinical trade-off. While Model 3 excels at detecting Moderate lesions based on color biomarkers, RICNN or Model 1 (Gabor + CNN) offers the most robust safety net for preventing blindness. Its capability to maintain near-perfect classification for Proliferative DR (99% AUC) makes it the most viable candidate for a clinical decision support system, where the priority is to ensure that advanced, vision-threatening cases are never missed.

#### 4.3.2. Impact of Class Imbalance Handling (SMOTE)

Beyond feature extraction, the distribution of class labels significantly influences classification performance in diabetic retinopathy analysis. In the present study, SMOTE was incorporated during the training phase to alleviate the effects of class imbalance across disease severity levels. This approach supports more balanced learning by increasing the representation of underrepresented classes and reducing bias toward dominant categories.

Despite these benefits, it is recognized that synthetic oversampling methods may introduce artificial feature variations that do not fully correspond to genuine pathological patterns observed in clinical retinal images. To limit potential effects on clinical reliability, SMOTE was applied exclusively to the training data, and all reported performance metrics were obtained from validation and test sets composed solely of real retinal images. The possible influence of synthetic samples on model interpretability is therefore acknowledged as a limitation, and future studies will investigate alternative data-balancing strategies that better align with clinically realistic variations.

#### 4.3.3. Deployment Considerations

While a detailed quantitative evaluation of inference latency was not conducted in this study, the computational properties of the proposed framework indicate its potential suitability for deployment in clinical environments supported by standard computing infrastructure. In particular, the Moderate model size and the offline execution of Gabor-based feature extraction contribute to manageable computational requirements during inference, which is governed solely by the CNN forward pass. Nevertheless, comprehensive runtime benchmarking and further model optimization were beyond the scope of the present work and will be addressed in future studies, particularly to support real-time or resource-limited clinical applications.

## 5. Conclusions

This study evaluated the efficacy of three distinct feature extraction strategies integrated with Convolutional Neural Networks (CNNs) for the multi-grade classification of diabetic retinopathy. The experimental results demonstrate that the proposed RICNN or Model 1 (Gabor + CNN) achieves superior performance compared to the Local Binary Pattern (Model 2) and Color Histogram (Model 3) baselines. Specifically, the RICNN attained an overall accuracy of 89%, an F1-score of 89%, and a remarkable AUC of 99% for Proliferative DR. These results indicate that Gabor-based feature extraction effectively captures directional vascular patterns such as neovascularization and vessel tortuosity, which are important for advanced diabetic retinopathy classification.

The Color Histogram-CNN (Model 3) is effective at detecting Moderate-stage lesions, such as hemorrhages and exudates, due to their distinct chromatic features; however, it shows limitations in representing complex vascular structures in the Proliferative stage. Similarly, the LBP-CNN (Model 2) provides stable but limited performance due to insufficient global directional sensitivity for extended vascular abnormalities. These findings suggest that relying solely on color or micro-texture features is insufficient for comprehensive diabetic retinopathy grading, and that structural information is also important.

From a clinical perspective, the RICNN’s high sensitivity to Severe and Proliferative DR stages makes it a promising approach for supporting early detection of vision-threatening conditions. The integration of SMOTE balancing and Adam optimization contributes to improved class balance and stable training. Future work will explore hybrid feature integration (e.g., combining Gabor and color information) and validation on multi-center datasets to improve generalization in real-world computer-aided diagnosis systems.

## Figures and Tables

**Table 1:** Feature extraction method and feature dimensions.

Feature Extraction Method	Parameters	Feature Dimensions
Gabor Filter	Frequencies = {2, 1.9, 1.7, 1.5, 1.3, 1, 0.9, 0.7, 0.5} Orientations = $\left\{0{}^{\circ},\;30{}^{\circ},45{}^{\circ},60{}^{\circ},90{}^{\circ}\right\}$ Image resize = 224 × 224	50,176
LBP	Uniform LBP, P = 8, R = 1, histogram-based	10
Color Histogram	RGB, 32 bins per channel	96
Total	-	50,282

**Table 2:** RICNN architecture technical specifications.

Parameters	Detailed Specifications
Layers	13 main layers (2 Conv, 2 Pooling, 1 Flatten, 2 Dense, 3 Dropout, 3 Batch Normalization)
Input Size	224 × 224
Convolution Layers	Layer 1: 32 filters, Layer 2: 64 filters
Kernel Size	3 × 3 for all convolution layers
Strides	1 (default value in Keras/TensorFlow)
Pooling Type	2D Max Pooling with a 2 × 2 window size
Flattening Dimensions	186,624 (result of 54 × 54 × 64)
Dense Layer	2 layers (1 Hidden Layer with 128 neurons and 1 Output Layer)
Dropout	3 Dropout
Batch Normalization	3 Batch Normalization
Activation Function	ReLU (Hidden Layer) and Softmax (Output Layer)
Output Size	4 Classes (according to the level of diabetic retinopathy)

**Table 3:** Results of the fundus image classification experiment of diabetic retinopathy using the Gabor filter and CNN methods.

Class	Precision	Recall	F1-Score
Mild	84%	75%	80%
Moderate	98%	96%	97%
Severe	89%	94%	91%
Proliferate DR	84%	91%	88%
Accuracy			89%
Macro-Avg	89%	89%	89%
Weighted Avg	89%	89%	89%

**Table 4:** Results of the fundus image classification experiment of diabetic retinopathy using LBP and CNN methods.

Class	Precision	Recall	F1-Score
Mild	76%	86%	81%
Moderate	98%	91%	94%
Severe	84%	89%	87%
Proliferate DR	91%	80%	85%
Accuracy			86%
Macro-Avg	87%	86%	87%
Weight Avg	87%	86%	86%

**Table 5:** Results of the fundus image classification experiment of diabetic retinopathy using Color Histogram and CNN methods.

Class	Precision	Recall	F1-Score
Mild	72%	75%	73%
Moderate	96%	96%	96%
Severe	100%	88%	93%
Proliferate DR	83%	88%	86%
Accuracy			87%
Macro-Avg	88%	87%	87%
Weighted Avg	88%	87%	87%

**Table 6:** Comparison of the confusion matrix evaluation results in the diabetic retinopathy fundus image classification model experiment (in percent).

Model	Accuracy	Precision	Recall	F1-Score
CNN	36%	0.18%	0.22%	0.18%
Model 1 (Gabor + CNN)	89%	88.75%	89%	89%
Model 2 (LBP + CNN)	86%	87.25%	86.50%	86.75%
Model 3 (CH + CNN)	87%	87.25%	86.75%	87%

**Table 7:** Comparison of AUC ROC curve evaluation results in diabetic retinopathy fundus image classification model experiment (in percent).

Class	CNN	Model 1 (Gabor + CNN)	Model 2 (LBP + CNN)	Model 3 (CH + CNN)
Mild	47%	93%	94%	96%
Moderate	42%	100%	98%	100%
Severe	54%	97%	98%	98%
Proliferate DR	43%	99%	95%	95%

## Data Availability

The data presented in this study are available from the corresponding author upon reasonable request.
